# Spondyloocular Syndrome: A Novel *XYLT2* Variant with Description of the Neonatal Phenotype

**DOI:** 10.3389/fgene.2021.761264

**Published:** 2021-12-03

**Authors:** Gabriella Doddato, Alessandra Fabbiani, Chiara Fallerini, Mirella Bruttini, Theodora Hadjistilianou, Martino Landi, Caterina Coradeschi, Salvatore Grosso, Barbara Tomasini, Maria Antonietta Mencarelli, Alessandra Renieri, Francesca Ariani

**Affiliations:** ^1^ Medical Genetics, University of Siena, Siena, Italy; ^2^ Med Biotech Hub and Competence Center, Department of Medical Biotechnologies, University of Siena, Siena, Italy; ^3^ Genetica Medica, Azienda Ospedaliera Universitaria Senese, Siena, Italy; ^4^ Ophthalmological Science and Neuroscience, Azienda Ospedaliera Universitaria Senese, Siena, Italy; ^5^ Terapia Intensiva Neonatale, Azienda Ospedaliera Universitaria Senese, Siena, Italy; ^6^ Pediatria, Azienda Ospedaliera Universitaria Senese, Siena, Italy

**Keywords:** spondyloocular syndrome (SOS), xylosyltransferase II, exome sequencing (ES), skeletal dysplasia, *XYLT2*

## Abstract

Spondyloocular syndrome (SOS) is a skeletal disorder caused by pathogenic variants in *XYLT2* gene encoding a xylotransferase involved in the biosynthesis of proteoglycans. This condition, with autosomal recessive inheritance, has a high phenotypic variability. It is characterized by bone abnormalities (osteoporosis, fractures), eye and cardiac defects, hearing impairment, and varying degrees of developmental delay. Until now only 20 mutated individuals have been reported worldwide. Here, we describe two siblings from consanguineous healthy parents in which a novel homozygous frameshift variant c.1586dup p(Thr530Hisfs*) in the *XYLT2* gene was detected by exome sequencing (ES). The first patient (9 years) presented short stature with skeletal defects, long face, hearing loss and cataract. The second patient, evaluated at a few days of life, showed macrosomia, diffuse hypertrichosis on the back, overabundant skin in the retronucal area, flattened facial profile with drooping cheeks, elongated eyelid rims, wide and flattened nasal bridge and turned down corners of the mouth. During the prenatal period, high nuchal translucency and intestinal hyperechogenicity were observed at ultrasound. In conclusion, these two siblings with a novel pathogenic variant in *XYLT2* further expand the clinical and mutational spectrum of SOS.

## Introduction

Spondyloocular syndrome (SOS) (OMIM #605822) is a very rare autosomal recessive skeletal disorder caused by pathogenic variants in *XYLT2* gene located on chromosome 17q21 (Umair et al., 2018). The main features include generalized osteoporosis, multiple long bone and spinal compression fractures, platyspondyly, short stature, cataract, cardiopathy, sensorineural hearing loss and varying degree of intellectual disability (Alanay et al., 2006; Taylan et al., 2017).

The *XYLT2* gene encodes a xylosyltransferase II, which catalyzes the initial phase of proteoglycans (PGs) assembly. PGs are found on the cell surface, in secretory granules and in the extracellular matrix. PGs are essential for many physiological processes, such as signal transduction, cellular homeostasis, membrane integrity, corepressor activity, morphogen gradient formation, lipid catabolism and scaffolding (MisMis et al., 2014). PGs consist of a core protein, which is linked to glycosaminoglycan (GAG) disaccharide chains. Assembly of GAGs on the core protein results in different groups of sulfated PGs such as chondroitin sulfate (CSPGs), heparan sulfate (HSPGs) and modified form of CSPGs, the dermatan sulfate (DSPGs) (Couchman and Pataki, 2012).

SOS was first clinically described in 2001 by Schmidt and others in a consanguineous family with six affected children. Clinical features of these patients included cataract, loss of vision due to retinal detachment, facial dysmorphisms and hypotonia, normal height with disproportionate short trunk, immobile spine with thoracic kyphosis and reduced lumbar lordosis (Schmidt et al., 2001). Alanay et al. reported another case with SOS in a Turkish family confirming that the syndrome was a distinct clinical entity (Alanay et al., 2006). In 2015, whole exome sequencing revealed homozygous mutations in the *XYLT2* gene as the molecular basis of SOS (Munns et al., 2015). To date, only 20 individuals with XYLT2-deficiency have been described. We report on two additional siblings with a novel homozygous *XYLT2* mutation. One of them represents the youngest patient with SOS ever described. Fetal abnormalities were documented pinpointing hints for the prenatal diagnosis.

## Materials and Methods

### Sample end DNA extraction

DNA samples were obtained at the Medical Genetics Unit of the Azienda Ospedaliera Universitaria Senese (A.O.U.S., Siena, Italy) upon the signature of informed consent for both diagnostic and research purposes. Genomic DNA was extracted from EDTA peripheral blood samples using MagCore HF16 (Diatech Lab Line, Jesi, Ancona, Italy).

### Exome sequencing

Exome sequencing was performed on genomic DNA samples of the proband and both parents using the Life Technologies Ion Proton sequencer (Life Technologies, Carlsbad, CA, USA). This system enables >92% of bases covered ≥20×. Sample preparation and sequencing were performed with AmpliSeq Exome strategy, following the manufacturer’s protocol (Life Technologies). The library preparation was performed using the Ion AmpliSeq Exome Kit (Life Technologies), which allows us to target ∼33 Mb of coding exons plus 15 Mb of flanking regions for a total of ∼58 Mb, in total more than 97% of the coding regions, using 12 primer pools for highly specific enrichment of exons within the human genome. Taking advantage of a barcode system, three samples were loaded together in a single run and sequenced. Data analysis was performed with Torrent Suite Software v5.0.2 (Life Technologies). Using specific parameters, we were able to remove the adaptors’ contamination and low-quality sequences, so the total amount of clean data was mapped to the UCSC/hg19 reference genome. Indel and variant calls were made using GATK version 2.7 (Broad Institute, Cambridge, MA, USA) and then the variants were also annotated against external datasets, including 1000 genomes, ExAC, gnomAD database and dbSNP147.

### Sanger sequencing

The *XYLT2* variant was confirmed by Sanger sequencing of exon eight including flanking intron sequences of the gene (NM_022167) in the probands and both parents.

DNA was amplified by PCR using specific primer pairs (forward 5′-ACA​ACA​GCA​GCA​GGA​AAA​GC-3′; reverse 5′-CTT​CAG​ACT​GGG​GCC​TTG​TT-3′), PCR products were sequenced employing ABIPRISM3130 Genetic Analyzer (Applied Biosystems, Foster City, CA, USA) and data were analyzed with Sequencher software V.4.9 (Gene Codes, Ann Arbor, USA).

### Mutation nomenclature

The mutation is described according to Human Genome Variations Society (HGVS). Nucleotide numbers are derived from the cDNA sequence of *XYL*T2 (GenBank accession NM_022167).

## Results

### Clinical description

**FIGURE 1:**
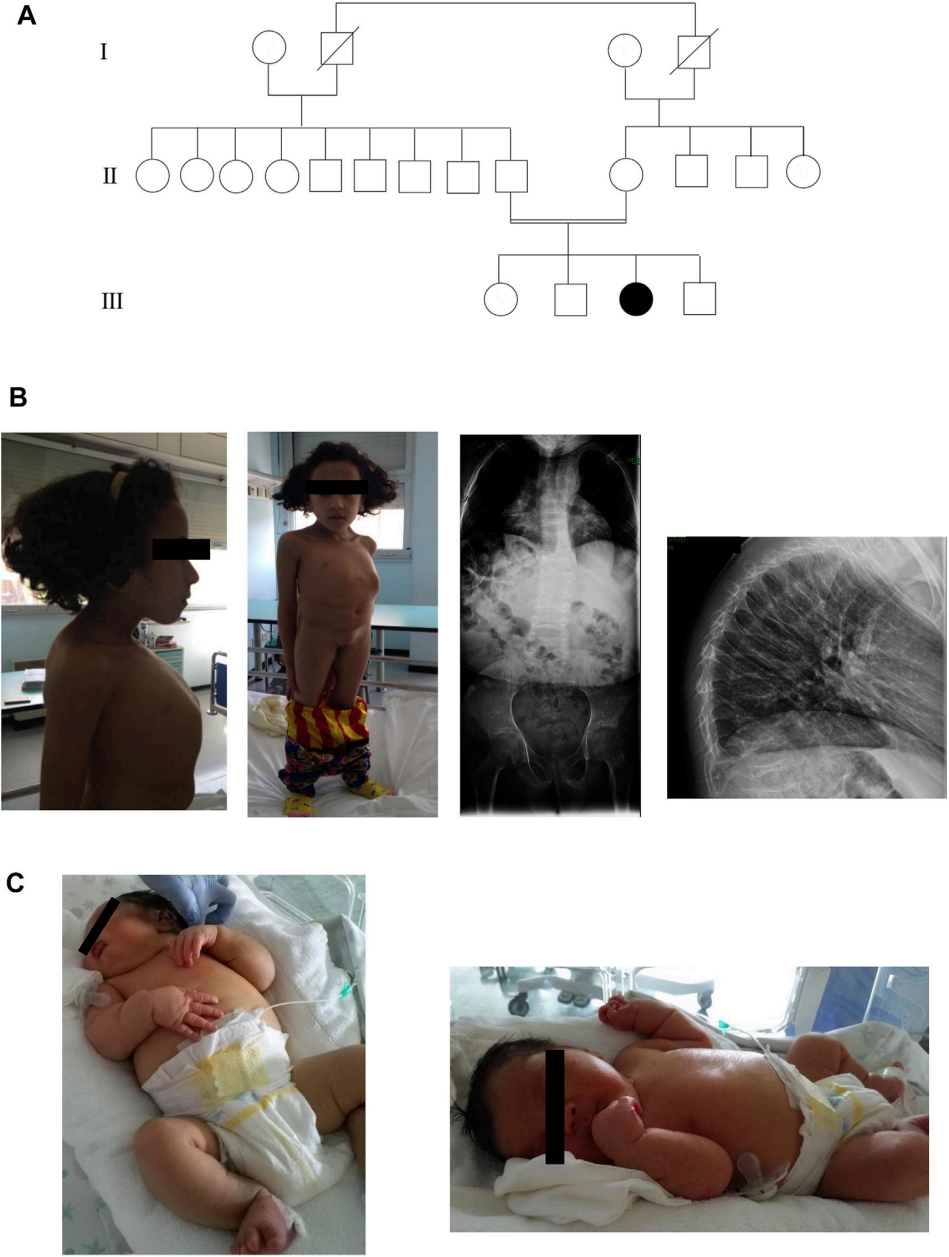
**(A)**Three generations of pedigree of family. **(B)** Anterior, lateral photographs and radiographs of sibling 1 that demonstrate platyspondyly with thinning of the vertebral bodies and secondary kyphosis. **(C)** Photographs of sibling 2 showing the main clinical features.

Here, we present two SOS affected siblings from consanguineous healthy parents of Moroccan origin (Figure 1A).

#### Sibling 1

Sibling 1 was evaluated for the first time in our Unit at the age of 9 years. We have little information about prenatal history. She was born from an uncomplicated pregnancy at full term. Her birth weight was 4,000 g (90° percentile). The baby showed hypovalid suction and poor growth. She had speech and psychomotor delay: she crawled at 5 years old, she was able to walk at 6 years old, she acquired sphincter control at 6 years old. She presented two fractures to the wrist and left foot after a fall. Physical examination revealed an occipital frontal circumference (OFC) of 51 cm (25th percentile), dental anomalies, short neck with pterygium, short stature with marked reduction in trunk length, severe kyphoscoliosis and barrel chest with increased intermammillary distance, in the absence of limb length reduction. In addition, she had long and oval face with flattened facial profile and blue sclerae, dorsal and pectoral hirsutism, generalized muscular hypotrophy. The instrumental examinations revealed platyspondyly with thinning of the vertebral bodies due to osteoporosis and thoracic deformity with secondary kyphosis and bilateral posterior subcapsular cataract and hearing loss (Figure 1B). At age of 13 years, biochemical analysis of patient’s blood revealed normal levels of calcium whereas serum 25-OH vitamin D level was decreased. Her ECG was normal, but she presented aneurysm of ascending aorta. The patient did not perform treatment with bisphosphonate.

#### Sibling 2

Sibling 2, evaluated at a few days of life, was born at 36 weeks of gestational age with a caesarean section due by oligohydramnios, generalized fetal edema, hydrops and macrosomia. Moreover, during pregnancy, fetal malformations and maternal diabetes were observed. Infectious investigation of the mother and fetus was carried out and the serologies were negative.

An ultrasound performed at 15 + 3 weeks of gestation revealed an increased nuchal translucency (6.5 mm), hyperechoic intestine, oligohydramnios, cystic hygromas with difficulties in visualizing the other viscera and cerebral hemispheres, a discrepancy between fetal biometry and age of reported amenorrhea. The parents refused to perform prenatal invasive diagnosis. The auxological parameters at birth were length, 49 cm (75th percentile); weight, 3,990 g (>97° percentile); and OFC, 34 cm (75th percentile). Apgar score was 6 at the first minute, 9 at the fifth minute and 10 at the tenth minute. Echocardiography carried out immediately after the birth showed mild hypertrophy of the left ventricle, endowed with mild-to-moderate global contractile dysfunction. Furthermore, left bovine arch with origin of left common carotid artery from right brachiocephalic trunk, mitral-aortic discontinuity with subaortic conus and dysplastic aortic valve were displayed. During hospitalization, the patient kept hemodynamic stability.

At the physical examination, we found: severe facial dysmorphic features such as flattened profile with drooping cheeks, elongated eyelid rims, broad and flattened nasal root and turned down corners of the mouth, chin with horizontal crease, macrosomia, diffuse hypertrichosis on the back and overabundant skin in the retronucal area. Furthermore, the baby showed hepatomegaly and minimal bilateral subdiaphragmatic fluid flap. The child underwent brain ultrasonograpy and metabolic test with normal results. An opalescent appearance of the cornea bilaterally was observed. The ophthalmologist evaluation highlighted a more remarkable corneal leucoma on the right eye. Topical anti-edema therapy was then performed showing a progressive improvement of the corneal edema. The patient underwent brain MRI, eye sockets and spine which get normal results. Furthermore, the auditory brainstem response (ABR) test found severe deafness. In addition, the baby presented right cryptorchidism (Figure 1C). Biochemical analysis of patient’s blood revealed normal levels of calcium and 25-OH vitamin D.

### Genetic analysis

**FIGURE 2:**
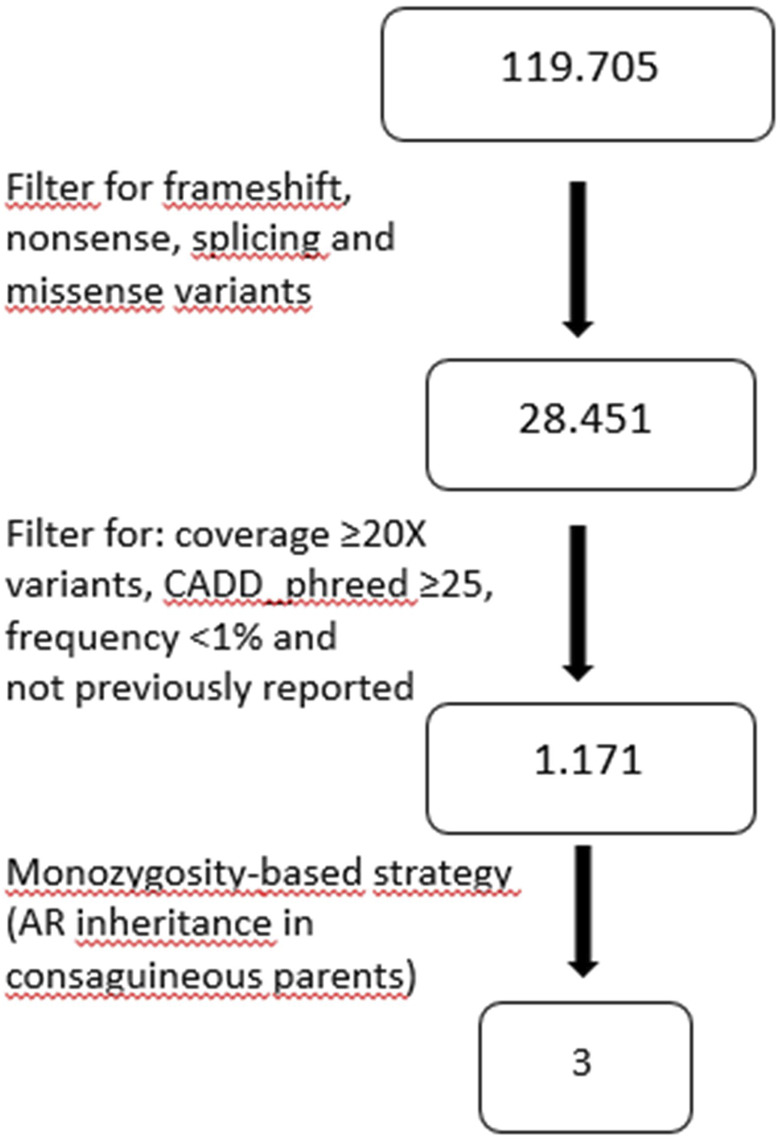
Workflow diagram of variants’ filtering in patient-parents trio exome.

**FIGURE 3:**
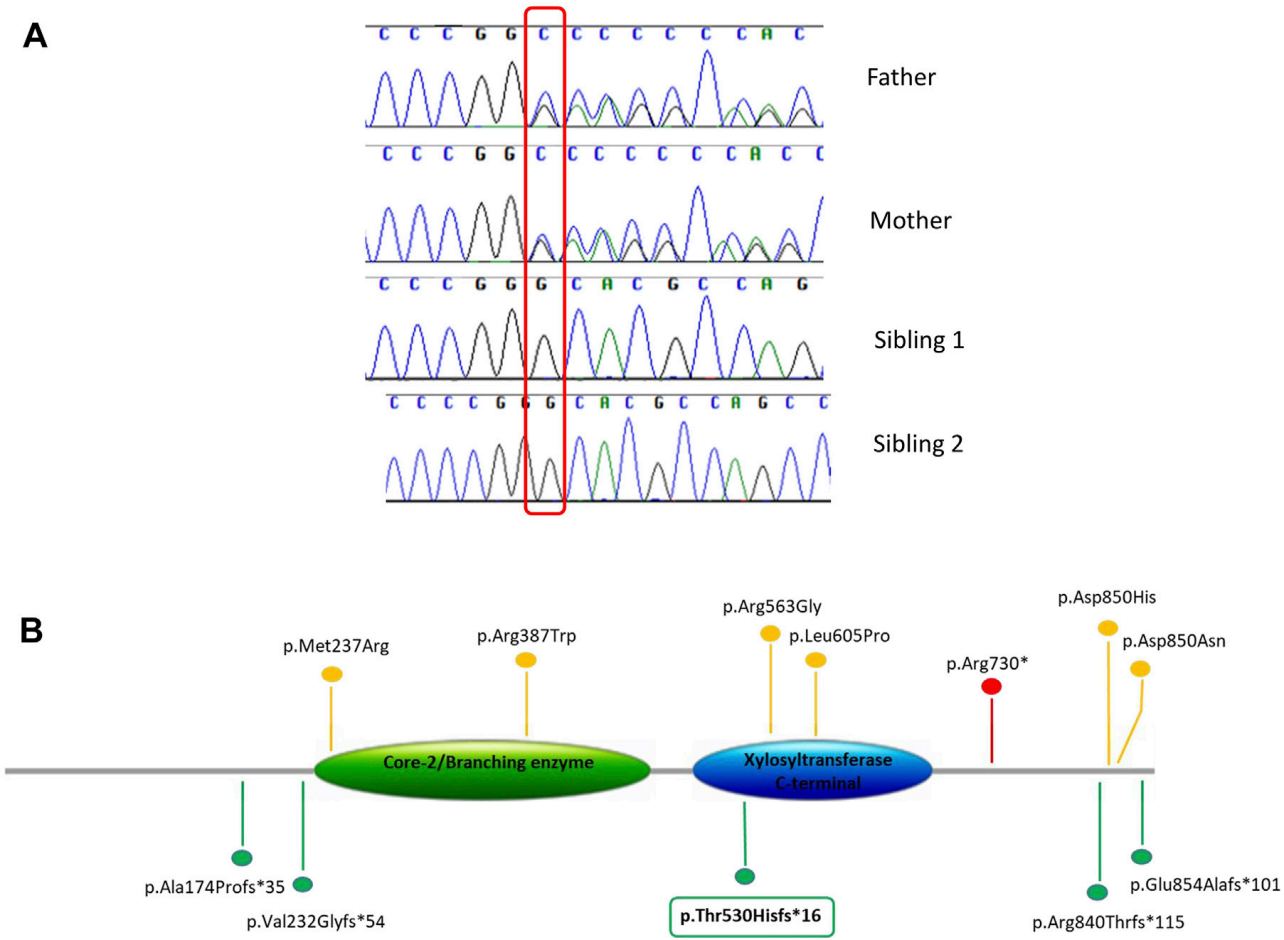
**(A)** Variant confirmation and segregation analysis by Sanger sequencing. **(B)** Schematic view of the XYLT2 protein (NM_022167), showing previously reported variants and the novel variant identified in this study (in box). Different types of variants are labeled with different colors.

Exome sequencing analysis was performed in sibling 1 and her parents. We obtained a mean depth of coverage of 91X. A total of 39,900 genetic variants on average for each sample was yielded. We filtered variants that were either loss-of-function (frameshift, nonsense and splicing variants) or missense variants (28,450 variants). Among them, variants with coverage ≥20X, variants CADD_phreed ≥25 (applied only to missense variants), frequency <1% or not reported were filtered. Considering the parental consanguinity, we decided to focus our attention on variants lying in disease genes consistent with autosomal recessive mode of inheritance (Figure 2). The analysis revealed a frameshift variant c.1586dup p.(Thr530Hisfs*16) in exon 8 of the *XYLT2* gene on chromosome 17. Sanger sequencing confirmed the homozygous variant in the proband and the heterozygous variant in both parents. In sibling 2, Sanger sequencing revealed the same mutation in the *XYLT2* gene in homozygous state (Figure 3A). This frameshift deletion has not been previously reported and it is absent in the ExAC or gnomAD database.

The variant has been submitted to the LOVD database (https://www.LOVD.nl) with the following ID: 0000272447.

## Discussion

In the present study, we described two additional patients with SOS and reported one novel homozygous variant, a frameshift deletion in the *XYLT2* gene c.1586dup p.(Thr530Hisfs*16). The variant was identified by exome sequencing and confirmed by Sanger sequencing in the two siblings while parents were heterozygous carriers, in accordance with an autosomal recessive inheritance pattern. To date, only 20 patients with *XYLT2* mutations have been reported worldwide. These two siblings with a novel pathogenic variant further expand the clinical and mutational spectrum of SOS.

The *XYLT2* gene, located on chromosome 17q21.3–q22, encodes an 865 amino acid protein that transport xylose molecules from the nucleoside diphosphate donor (UDP-xylose) to targeted serine molecules of the core protein during the synthesis of proteoglycans. The *XYLT2* is composed of four domains: an N terminus domain, a xylosyltransferase terminal domain (catalytic domain), a core2/I-branching enzyme domain, and a C-terminus domain (Umair et al., 2018). The identified variant is predicted to truncate the C-terminus but this has not been shown experimentally and a haploinsufficiency effect could not be ruled out. To date, only 11 homozygous variants including a nonsense, six missense, one frameshift duplication and three frameshift deletions in the *XYLT2* gene have been reported (Umair et al., 2018; Taylan et al., 2017; Munns et al., 2015; Guleray et al., 2019; Kausar et al., 2019; Taylan et al., 2016) (Figure 3B). The phenotypic severity of SOS is suggested to be associated with the mutation type, localization and domain, but there is no agreement on this point and no obvious genotype phenotype correlation can be made (Alanay et al., 2006; Taylan et al., 2017; Umair et al., 2018). Missense variants are not always correlated with a milder phenotype and different truncating variants are not associated with the phenotypic spectrum depending on the localization. Indeed, our patients show a severe phenotype even if the variant truncates only the C-terminus. There is high phenotypic variability and other factors in the genome could modulate the clinical spectrum.

Concerning the phenotype, consistent findings include skeletal dysplasia with short stature (13/22), low weight (9/22), multiple fractures (19/22), kyphosis (15/22), and facial dysmorphisms (14/22). Other, more frequent features were ocular problems (21/22) and hearing loss (14/22). Dental problems (3/22), cardiovascular defects (7/22) and neurodevelopmental delay (10/22) are variably present (Umair et al., 2018; Taylan et al., 2017; Munns et al., 2015; Guleray et al., 2019; Kausar et al., 2019; Taylan et al., 2016) (Table 1). In some patients, the treatment with bisphosphonate appears to have a positive effect on the clinical course, but the exact mechanism between bisphosphonate treatment and proteoglycan biosynthesis has not been established (Munns et al., 2015; Guleray et al., 2019).

**TABLE 1:** Clinical features of the present case compared with the previously reported patients with *XYLT2* variants

	Present study		Kausar et al. (2019)						Guleray et al. (2019)					Umair et al. (2017)*^1,6,10^								
Family	1		2						3					4								5
Patient	Pat. 1	Pat. 2	Pat. 1		Pat. 2		Pat. 3		Pat. 1		Pat. 2			Pat. 1			Pat. 2		Pat. 3	Pat. 4	Pat. 5	Pat. 6
Gender	F	M	M		M		M		F		M			M			M		F	M	M	M
Age (years)	9	Newborn	12		9		10		10		7			21			19		16	11	9	5
Ethnicity	Moroccan		Pakistani						Turkish					Iraqi								Turkish
Parental consanguinity	+		+						+					+			+		+	+	+	−
Variant	c.1586dup p.(Thr530Hisfs*16)		c.2518_2519del p.(Arg840Thrfs*115)						c.2548G > A p.(Asp850Asn)					c.1159C > T p.(Arg387Trp)								c.2548G > C p.(Asp850His)
Prenatal history	Uncomplicated pregnancy at full term	Fetal malformations Maternal diabetes Increased nuchal translucency Hyperechoic intestine	Uncomplicated pregnancy at full term		NA		NA		Pregnancy at full term No intrauterine fractures			Pregnancy at full term No intrauterine fractures		NA			NA		NA	NA	NA	Uncomplicated pregnancy with normal measurements at birth
Short stature	+	−	+		+		+		+				+	−			−		+	−	−	+
Low weight	+	−	+		+		+		+				+	−			−		−	+	−	+
Multiple fractures	+	−	+		+		+		+				NA	+			+		+	+	+	NA
Kyphosis	+	−	+		NA		+		+				+	+			+		+	+	+	+
Osteoporosis	+	−	+		NA		+		+				+	+			+		+	+	+	+
Cataract	+	−	+		+		+		+				+	+			+		+	+	+	+
Retinal detachment	−	−	+		−		−		+				−	NA			NA		+	+	+	−
Dental problems	+	−	−		−		−		−				+	−			−		−	−	−	−
Hearing loss	+	+	+		+		+		−				+	−			−		−	−	−	−
Cardiovascular problems	+	+	−		−		−		+				−	−			−		−	+	−	+
Facial dysmorphism	Long and oval face Flattened facial profile	Flattened profile with drooping cheeks Elongated eyelid rims Broad and flattened nasal root Turned down corners of the mouth Chin with horizontal crease Macrosomia	Low posterior hairline Short and webbed neck Low set ears Shield chest Long fingers and toes		NA		NA		High forehead Short neck Facial hypotonia				Epicanthal folds Facial hypotonia	Hypertelorism Facial hypotonia			Facial hypotonia		Hypertelorism Facial hypotonia Low nasal bridge Prognatism	Hypertelorism Facial hypotonia Low nasal bridge Prognatism	Hypertelorism Facial hypotonia Low nasal bridge Prognatism	Facial hypotonia Long philtrum with thin upper lip Low set ears with thick helices Anti-helix abnormality Preauricular pits
Ophthalmologic findings	Sclerae blue	NA	Nystagmus Amblyopia		−		−		Nystagmus Myopia Strabism Grayish sclerae				Grayish sclerae	Micropthalmia Leucoma cornea Phtisis bulbi			Hyperemic optic nerve Thin retinal vessels and partially occluded Retinal atrophy Pigment irregularities		Band keratopathy Congenital melanosis Pigment irregularities Thin retinal vessels	Pigment epithelium Atrophy of retina	Micropthalmia Retinal atrophy Pigment irregularities	Nystagmus and myopia Hyperpigmented and hypopigmented retinal areas Thin retinal vessels
Neurodevelopmental delay	Intellectual disability	NA	Learning difficulties		Learning difficulties		Learning difficulties		−				Intellectual disability	−			−		−	−	−	Intellectual disability/learning difficulties
Joint and skin elasticity	−	−	−		−		−		−				−	−			−		−	−	−	+
Walking difficulty	−	−	+		−		NA		+				+	NA			NA		NA	NA	NA	+
Other clinical findings	Short neck with pterygium Reduction in trunk length Severe kyphoscoliosis Barrel chest	Diffuse hypertrichosis on the back Overabundant skin in the retronucal area Cryptorchidism Hepatomegaly	NA		NA		Low posterior hairline Short and webbed neck Low set ears Shield chest Long fingers and toes		Short trunk Lordosis Mild pes planus Varicose vein Hepatomegaly Truncal obesity				Pes planus Enlarged liver and spleen Truncal obesity	Pes planus Broadened fingertips			Pes planus Broadened fingertips		Pes planus Broadened fingertips	Pes planus Broadened fingertips	Pes planus Broadened fingertips	Pes planus Short trunk
Other radiographic findings	Platyspondyly Thoracic deformity with secondary kyphosis	NA	Platyspondyly Increased intervertebral disk space		NA		Platyspondyly		Platyspondyly Intervetebral disc space Increased intervertebral disc space				Platyspondyly	Platyspondyly Scoliosis			Platyspondyly Scoliosis		Platyspondyly Scoliosis	Platyspondyly Scoliosis	Platyspondyly Scoliosis	Platyspondyly Increase in intervertebral disc space

	Taylan et al. (2017)			Taylan et al. (2016)											Munns et al. (2015)							
Family	6	7		8		9				10					11			12				
Patient	Pat. 1	Pat. 2		Pat. 1		Pat. 2		Pat. 3		Pat. 4					Pat. 1	Pat. 2		Pat. 3				
Gender	F	F		F		F		M		M					M	M		M				
Age (years)	7	7		8		16		7		11					14	11		19				
Ethnicity	Turkish	Turkish		Turkish		Pakistani				Iraqi					European Australian			NA				
Parental consanguinity	−	+		+		+		+		+					−	−		+				
Variant	c.710T > G p.(Met237Arg)	c.2561_2562del p.(Glu854Alafs*101)		c.2188C > T p.(Arg730*)		c.1687C > G p.(Arg563Gly)				c.1814T > C p.(Leu605Pro)					c.692dup p.(Val232Glyfs*54)			c.520del p.(Ala174Profs*35)				
Prenatal history	Pregnancy at full term No intrauterine fractures Normal birth measurements	NA		NA		NA		NA		NA					Born at 39 weeks	NA		Born prematurely 35 weeks				
Short stature	+	+		+		−		−		+					−	−		+				
Low weight	+	NA		NA		−		−		−					−	−		+				
Multiple fractures	+	+		+		+		+		+					+	+		+				
Kyphosis	+	−		+		+		+		NA					NA	NA		NA				
Osteoporosis	+	+		+		+		+		NA					NA	NA		NA				
Cataract	+	+		+		+		+		+					+	+		+				
Retinal detachment	−	−		−		−		−		−					+	−		+				
Dental problems	−	−		−		+		−		−					−	−		−				
Hearing loss	+	+		+		+		−		+					+	+		+				
Cardiovascular problems	−	−		+		−		−		−					+	+		−				
Facial dysmorphism	Minimal biparietal bossing Upslanted short palpebral fissure	Proptosis Prominent cheeks Synostosis of metopic suture		NA		NA		NA		NA					Low posterior hairline Short webbed neck Posteriorly rotated and low set ears	NA		NA				
Ophthalmologic findings	−	Iris hypoplastic Pupils irregular Peripapillary atrophy Hypopigmented fundus		Nystagmus Bilateral glaucoma		Ectropion uvea		−		Nystagmus					Amblyopia Nystagmus	−		−				
Neurodevelopmental delay	−	Intellectual disability		Intellectual disability		NA		NA		NA					Learning difficulties	Learning difficulties		−				
Joint and skin elasticity	−	−		−		−		+		−					−	−		−				
Walking difficulty	NA	−		NA		NA		NA		NA					+	−		+				
Other clinical findings	Pectus excavatum	Pectus excavatum		Aggressive behavior		Translucent appearing teeth		Spontaneous fracture of teeth		Pectus carinatum					Lymphedema of lower extremities Perforated duodenal ulcer Pes planus pectus carinatum, shield chest Long finger Overriding second and third toes Dilated ureters	Pes planus		Pes planus Mild left hemiplegia				
Other radiographic findings	Short spine	−		Short trunk Osteopenia Platyspondyly Increased disc space		Anterior wedge deformity Biconcave appearance of the vertebral and plates		−		Short spine					−	Thin cortices of hand and wrist		Thin cortices of hand and wrist				

NA, not available; M, male; F, female; +, present; −, absent. *Patients described by Schmidt et al. (2001) and Alanay et al. (2006).

We described the youngest patient affected by SOS with important hints for prenatal diagnosis: increased nuchal translucency, hyperechoic intestine, oligohydramnios and cystic hygromas. The phenotype of the newborn includes macrosomia, diffuse hypertrichosis on the back and overabundant skin in the retronucal area. He also displayed severe facial dysmorphic features. Moreover, minor cardiac abnormalities, ocular alteration and hearing loss were in place.

In conclusion, we have reported a novel frameshift deletion in two additional patients with SOS. The variant is a truncating deletion that disrupts the C-terminus domain of the protein. These findings extend the mutation spectrum of *XYLT2-*related disorders and help proper genotype–phenotype correlation.

## Data Availability

The datasets for this article are not publicly available due to concerns regarding participant/patient anonymity. Requests to access the datasets should be directed to the corresponding author.
