# Corrigendum: Spondyloocular syndrome: A novel *XYLT2* variant with description of the neonatal phenotype

**DOI:** 10.3389/fgene.2023.1143795

**Published:** 2023-01-25

**Authors:** Gabriella Doddato, Alessandra Fabbiani, Chiara Fallerini, Mirella Bruttini, Theodora Hadjistilianou, Martino Landi, Caterina Coradeschi, Salvatore Grosso, Barbara Tomasini, Maria Antonietta Mencarelli, Alessandra Renieri, Francesca Ariani

**Affiliations:** ^1^ Medical Genetics, University of Siena, Siena, Tuscany, Italy; ^2^ Med Biotech Hub and Competence Center, Department of Medical Biotechnologies, University of Siena, Siena, Tuscany, Italy; ^3^ Genetica Medica, Azienda Ospedaliera Universitaria Senese, Siena, Tuscany, Italy; ^4^ Ophthalmological Science and Neuroscience, Azienda Ospedaliera Universitaria Senese, Siena, Tuscany, Italy; ^5^ Terapia Intensiva Neonatale, Azienda Ospedaliera Universitaria Senese, Siena, Tuscany, Italy; ^6^ Pediatria, Azienda Ospedaliera Universitaria Senese, Siena, Tuscany, Italy

**Keywords:** spondyloocular syndrome (SOS), xylosyltransferase II, Exome Sequencing (ES), skeletal dysplasia, *XYLT2*

In the published article, there was an error. There was a sentence missing in the **Acknowledgments** section.

A correction has been made to the **Acknowledgments**. This sentence previously stated:

“The authors thank the family for participating in the study.”

The corrected sentence appears below:

“The authors thank the family for participating in the study. Two of the authors of this publication are members of the European Reference Network for rare malformation syndromes and rare intellectual and neurodevelopmental disorders, ERN-ITHACA.”

The authors apologize for this error and state that this does not change the scientific conclusions of the article in any way. The original article has been updated.

